# Cytoreduction and Hyperthermic Intraperitoneal Chemotherapy for Pseudomyxoma Peritonei of Appendiceal Origin: A Single Center Experience

**DOI:** 10.3389/fsurg.2021.715119

**Published:** 2021-08-26

**Authors:** Francesco Santullo, Fabio Pacelli, Carlo Abatini, Miriam Attalla El Halabieh, Giusy Fortunato, Claudio Lodoli, Francesco Giovinazzo, Stefano Rotolo, Andrea Di Giorgio

**Affiliations:** ^1^Surgical Unit of Peritoneum and Retroperitoneum, Fondazione Policlinico Universitario A. Gemelli IRCCS, Rome, Italy; ^2^Departement of Anesthesiology and Intensive Care Medicine, Fondazione Policlinico Universitario A. Gemelli IRCCS, Rome, Italy; ^3^General Surgery and Liver Transplant Unit, Fondazione Policlinico Universitario A. Gemelli IRCCS, Rome, Italy; ^4^Department of Surgical, Oncological and Oral Sciences, University of Palermo, Palermo, Italy

**Keywords:** pseudomixoma peritonei, cytoreductive surgery, hyperthermic intra peritoneal chemotherapy, HIPEC, peritonectomy procedure

## Abstract

**Background:** Pseudomyxoma peritonei (PMP) originating from appendiceal mucinous neoplasm is a rare peritoneal malignancy characterized by the progressive intraperitoneal accumulation of mucus leading to death if left untreated. In recent years, cytoreductive surgery (CRS) combined with hyperthermic intraperitoneal chemotherapy (HIPEC) offered increased survival rates. This study aims to identify the clinical, pathological, and surgical features influencing safety and survival outcomes of patients undergoing CRS and HIPEC for PMP of appendiceal origin.

**Methods:** A retrospective analysis of all patients undergoing CRS and HIPEC for PMP of appendiceal origin from January 2015 to May 2019 was conducted at our institution.

**Results:** Study population included 50 patients (74% female, 26% male). The median age at CRS was 60 (38–84). The median peritoneal cancer index (PCI) was 17. Complete cytoreductive surgery (CC 0–1) was achieved in 47 patients (94%). HIPEC chemotherapeutic regimen was based on oxaliplatin for 13 (28%) patients and mitomycin for 34 (72%) patients. We experienced a total of 19 (38%) postoperative complications, of which 14 (74%) of grade I-II and 5 (26%) of grade III-IV, according to the Clavien-Dindo classification. The median follow-up period was 27 months (12–107) from the date of cytoreductive surgery. The mean survival rate was 100 months, with a 5-year OS of 91%. The mean progression-free survival rate was 77 months (0–107), with a 5-year PFS of 63%. Multivariate analysis identified adenocarcinoma histotype and incomplete cytoreduction to significantly worsen progression-free survival, while incomplete cytoreduction was the only independent predictor of poorer overall survival.

**Conclusion:** Complete cytoreduction and appendiceal neoplasm histotype play a crucial role in the survival of patients affected by PMP of appendiceal origin. The rates of morbidity associated with CRS and HIPEC for PMP are acceptable.

## Introduction

Pseudomyxoma peritonei (PMP) is a rare clinical condition characterized by the collection of free or organized mucin with or without neoplastic cells and a typical pattern of redistribution. The reported incidence is ~1 per million/year (1) and it is strongly related to appendiceal neoplasm, despite PMP may arise, less frequently, from mucinous tumors of the ovary, colon, or biliary tract (2). Appendiceal mucinous neoplasms are oftentimes low-grade and rarely associated with an extra-abdominal spread (2, 3).

Due to its indolent nature, PMP has a long natural history and grows undisturbed until the slow accumulation of mucin within the abdominal cavity leads to dyspnea, bowel obstruction, and eventually death.

In patients with PMP, CRS with HIPEC is considered a potentially curative treatment in several nationals and clinical societies guidelines (3, 4), with a good risk-benefit ratio in selected patients, despite clear evidence is lacking. This combined approach has been reported to allow a 15-year survival rate of 59%, and a progression-free survival of 8.2 years (5). However, CRS and HIPEC are associated with non-negligible morbidity and mortality and understanding the value of preoperative and procedure and disease-related factors may help in patient selection and counseling.

The main objective of the study is to identify clinical, pathological, and surgical features that may influence the safety, the progression-free and the overall survival of PMP of appendicular origin treated with CRS and HIPEC.

## Patients and Methods

The present study is a retrospective analysis of patients undergoing CRS and HIPEC for PMP of appendicular origin at our institution between January 2015 and May 2019. Relapse and survival data were collected and stored in a prospectively maintained database. The study received the approval of the local Institutional Review Board.

All patients with suspected PMP received a detailed physical examination, along with an accurate history, routine blood counts, as well as thorax, abdomen, and pelvic CT scans. A multidisciplinary meeting involving dedicated peritoneal surgeons, radiologists, and medical oncologists reviewed all cases. The surgical intent was to obtain a maximal cytoreduction and perform HIPEC. Hence, the burden of disease and the number of resections required were carefully estimated preoperatively. In case of doubts, the extent of peritoneal disease was assessed by laparoscopy through the peritoneal carcinomatosis index (PCI) scoring system. CRS was performed using the Sugarbaker technique (6). The decision to perform a total or a partial peritonectomy was made case by case depending on localization and disease load. At the end of CRS, HIPEC was carried out with the closed technique. In 7 cases, the HIPEC procedure was performed using the CO2 technology (7–9). The advantage of this technique is to obtain a more homogeneous drug distribution through turbulence brought by CO2 infusion into the abdominal cavity. Patients who could not achieve a complete cytoreduction, generally due to the extent of disease or their general conditions, underwent a maximal tumor debulking without HIPEC.

### Data

We extracted the following data from medical records: general demographic information, clinical presentation, date of diagnosis, preoperative markers and laboratory values, Eastern Cooperative Oncology Group (ECOG) performance score, histology of the appendiceal neoplasm, any preoperative chemotherapy regimen. Any previous surgery for the treatment of PMP was reported using the prior surgical score (PSS).

Residual disease was recorded using the four-tier completeness of cytoreduction (CC) score system. CC-0 corresponds to no macroscopic residual localizations. CC-1 indicates persisting localizations up to 2.5 mm in diameter, CC-2 residual nodules between 2.5 and 2.5 cm, and CC-3 residual disease >2.5 cm. CC0 and CC1 were regarded as complete cytoreduction, while CC2 and CC3 as incomplete cytoreduction.

Postoperative complications were scored using the Clavien-Dindo Classification (10). To perform statistical analysis, these were further subdivided into minor complications, corresponding to grade I and II, and major ones, corresponding to grade III and IV.

### Pathological Evaluation

According to the Peritoneal Surface Oncology Group International (PSOGI) classification (4), all cases were pathologically reviewed by a pathology with expertise in peritoneal surface malignancies and divided into four groups: patients with *acellular mucin only*, defined as mucin without neoplastic epithelium, either confined to the vicinity of the organ of origin or distant from it; patients with a *low-grade disease* (or disseminated peritoneal adenomucinosis, DPAM), defined as the presence of abundant extracellular mucin with scant strips or small islands (<20% tumor volume) of simple to focally proliferative epithelium, with minimal cytologic atypia and rare mitoses, patients with a *high-grade disease without signet ring cells* (or peritoneal mucinous carcinomatosis, PMCA), defined as more abundant cellularity (>20% tumor volume) and presence of one or more among high-grade cytology, infiltrative invasion into adjacent tissue, angiolymphatic or perineural invasion, and cribriform growth, patients with *high-grade disease and signet ring cells*, defined simply by the presence of the latter, generally when they are >10% of the cells.

### Statistical Analysis

Descriptive statistics were used to describe patient, surgical, and pathological characteristics. Continuous variables were reported as medians or means with the range, while categorical variables as numbers and percentages of the total group. Survival analysis was performed using the Kaplan–Meier method. Cox univariate analysis was utilized to evaluate associations between individual variables and PFS and OS.

Multivariate analysis was performed using the Cox proportional hazards model selected in a backward stepwise (likelihood ratio) regression. A significance level of 0.100 was used for the entry of variables in the multivariable model. *P* ≤ 0.050 was considered statistically significant.

Statistical analyses were performed using SPSS® version 24.0 (IBM, Armonk, New York, USA) software for Windows and Microsoft® Excel®.

## Results

### Patients' Characteristics

The internal database search retrieved 241 patients who underwent surgery for peritoneal surface malignancies of which 50 patients with a diagnosis of PMP of appendicular origin. Their demographic, clinical, and pathological characteristics are summarized in Table 1.

**Table 1:** Demographic, clinical, and pathological characteristics of the study population.

**Variable**	***N* (%)**
**Gender**	
Males	13 (26)
Females	37 (74)
**Age at CRS**	
Median	60
Range	38–84
**BMI**	
Median	25.6
Range	19.5–41.6
**ECOG score**	
0	33 (66)
≥1	16 (34)
**Prior surgical score (PSS)**	
0	17 (34)
1	18 (36)
2	10 (20)
3	5 (10)
**Preoperative chemotherapy**	
No	46 (92)
Yes	4 (8)
PSOGI classification	
Acellular mucin	10 (20)
Low-grade PMP (DPAM)	34 (68)
High-grade PMP (PMCA)	6 (12)
**Appendiceal histotype**	
LAMN	44 (88)
Adenocarcinoma	6 (12)

*CRS, cytoreductive surgery; BMI, body mass index; LAMN, low-grade appendiceal mucinous neoplasm; DPAM, disseminated peritoneal adenomucinosis; PMCA, peritoneal mucinous carcinomatosis*.

Study population included 37 (74%) female and 13 (26%) male patients. The median age at CRS was 60 (38–84). The median preoperative BMI was 25.6. According to the ECOG score, 33 patients (66%) had a score of 0, while 16 (33%) had a score of 1 or higher. The appendiceal histotype was low-grade appendiceal mucinous neoplasm (LAMN) in 44 (88%) patients and mucinous adenocarcinoma in the remaining 6 (12%). Considering peritoneal disease pathological analysis, according to the PSOGI classification, 10 (20%) patients presented acellular mucin only, 34 (68%) had a low-grade peritoneal disease or DPAM, and 6 (12%) had high-grade disease or PMCA. No one presented with a signet ring cell histology. The six patients (12%) with the high-grade disease received oxaliplatin-based chemotherapy preoperatively.

### Procedure-Related Outcomes and Safety

The median PCI score was 17. According to the CC score, 37 (74%) patients presented no residual disease (CC-0) and 10 (20%) had residual disease <0.25 cm (CC-1). These 47 (90%) patients received HIPEC. HIPEC was not administered to 3 (6%) patients with a residual disease over 0.25 cm (CC 2-3). The critical element precluding a complete cytoreduction and HIPEC in these three patients were extensive mesenteric and small bowel involvement and multiple comorbidities. After surgery, two of them received palliative care, and one received palliative oxaliplatin-based systemic chemotherapy. HIPEC chemotherapeutic regimen was oxaliplatin-based for the first 13 (28%) patients. After May 2018, we administered mitomycin 35 mg/m^2^ for the remaining 34 (72%) patients. The closed abdomen technique was used in all cases of which 7 (15%) with the CO_2_ technology.

Considering the surgical procedures carried out during CRS, patients most commonly received pelvic peritonectomy (86%), followed by omentectomy (84%) and diaphragmatic peritonectomy (82%). Thirty-seven patients (74%) received a total peritonectomy. The number of anastomoses ranged from 0 to 3, with one anastomosis per patient on average. Seventeen (34%) patients required a protective ileostomy. The mean operating time was 587 min. Mean hospital stay was 12 days (3–28). Surgical and perioperative data are summarized in Tables 2, 3.

**Table 2:** Perioperative data.

**Variable**	***N* (%)**
**PCI score**	
Median [range]	17 [3-34]
**Operating time**	
Median [range]	587 vs 683 [360-816]
**Completeness of cytoreduction (CC)**	
CC 0	37 (74)
CC 1	10 (20)
CC 2–3	3 (6)
**HIPEC**	
Yes	47 (94)
No	3 (6)
**HIPEC drugs**	
Oxaliplatin	13 (28)
Mitomycin C	34 (72)

**Table 3:** Operative details.

**Surgical procedures**	***N (% of patients)***
Total peritonectomy	37 (74)
Parietal peritonectomy	40 (80)
Pelvic peritonectomy	43 (86)
Diaphragmatic peritonectomy	41 (82)
Hysterectomy/oophorectomy	24 (48)
Mesenteric cytoreduction	4 (8)
Omentectomy	42 (84)
Bursectomy	39 (78)
Gastric resection	4 (8)
Small bowel resection	2 (4)
Splenectomy	30 (60)
Colon resection	24 (48)
Rectal resection	18 (36)
Cholecystectomy	27 (54)
Lymphadenectomy	4 (8)
Ostomy	17 (34)

We experienced a total of 19 (38%) postoperative complications (Table 4), of which 14 (74%) of grade I-II (minor complications) and 5 (26%) of grade III-IV (major complications), according to the Clavien-Dindo classification. None of these complications were due to HIPEC or HIPEC-CO_2_ procedures. One patient (2%) died within 30 days due to multiple colonic perforations and generalized sepsis.

**Table 4:** Postoperative complications.

**Variable**	***N* (%)**
**Complications**	
Yes	19 (38)
No	31 (62)
**Complication grade (Clavien-Dindo)**	
Grade I-II	14 (74)
Grade III-IV	5 (26)
Postoperative mortality	1 (2)
Postoperative ileus	3 (6)
Pulmonary complications	4 (8)
Postoperative bleeding	2 (5)
Abdominal collection	3 (6)
Genitourinary infection	3 (6)
Surgical site infection	4 (8)

### Recurrence and Survival Outcomes

**Figure 1:**
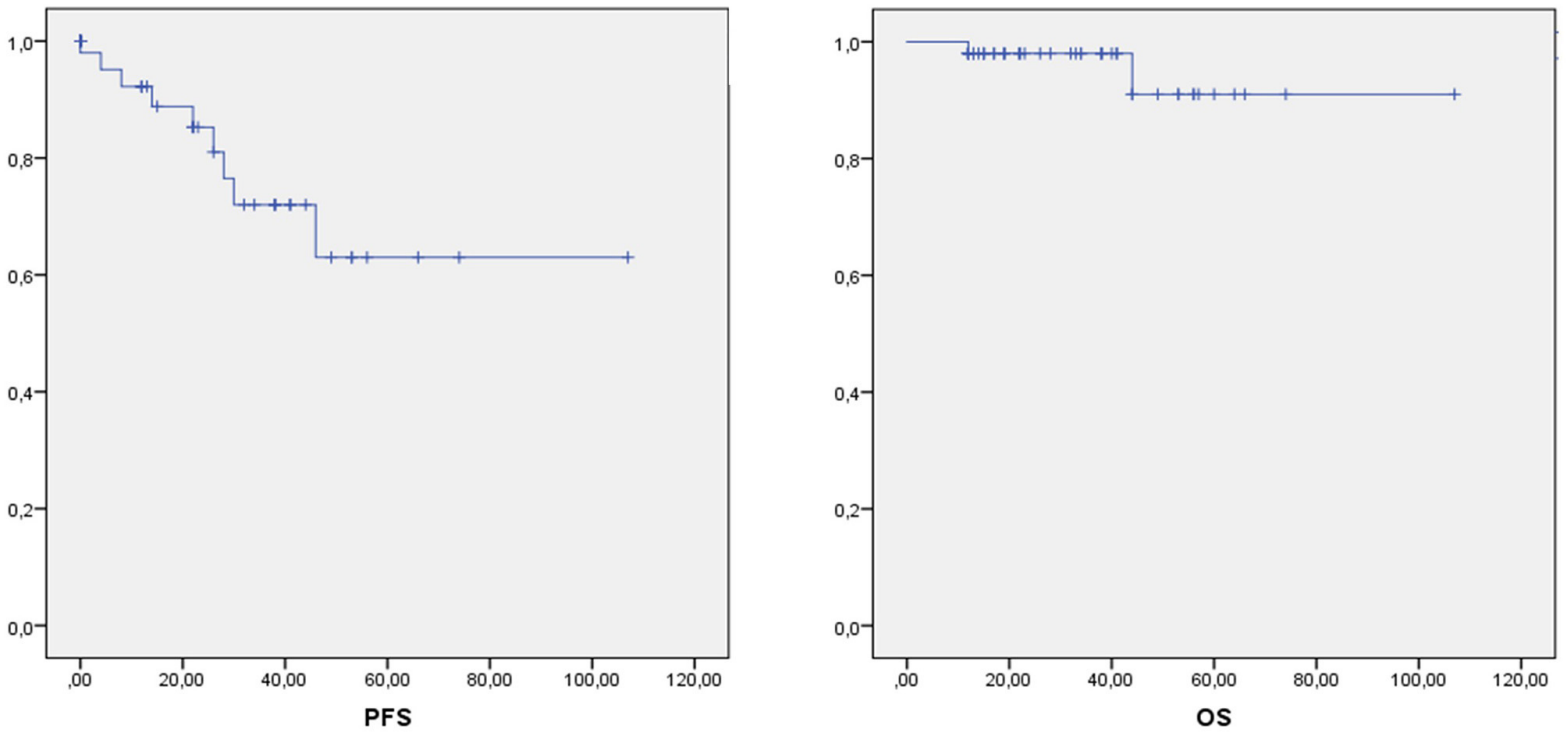
Progression free survival (PFS) and Overall Survival in our series.

The median follow-up period was 27 months from the date of cytoreductive surgery. The median progression-free survival was 77 months, with a 5-year PFS of 63% (Figure 1).

The recurrence rate in our series was 18%, 9 out of 50 patients. Recurrence was inside the abdominal cavity in 7 patients, thoracic in 3 cases, and at the level of the abdominal wall in another one. Two patients had a relapse both in the abdomen and in the thorax. Five patients out of nine received optimal secondary CRS and HIPEC: of these, 3 are currently free of disease, 1 had an abdominal recurrence, further treated with systemic chemotherapy and 1 is free of abdominal disease but developed thoracic localization. In particular, this patient received suboptimal cytoreduction of the pleural and pulmonary localizations plus Hyperthermic Intraoperative Thoracoabdominal Chemotherapy (HITOC) and is currently receiving systemic chemotherapy. One patient out of nine received only palliative HIPEC and systemic chemotherapy because of microscopic diffuse mesenteric recurrence. Considering the remaining three patients, one experienced an isolated recurrence of the abdominal wall and underwent surgical excision. He is currently free of disease. Two patients received only systemic oxaliplatin-based chemotherapy and then palliative care.

The median overall survival was 100 months, with a 5-year OS of 91% (Figure 1). At the time of this study, two patients (4%) have died. At univariate analysis, adenocarcinoma histotype of the appendiceal neoplasm (HR: 10.986, 95% CI: 2.555–47.238, *p* = 0.001), high-grade PMP (PMCA) (HR: 9.126, 95% CI: 2.385–34.914, *p* = 0.001) and incomplete cytoreduction (CC2-3) (HR: 4.985, 95% CI: 0.855–29.069, *p* = 0.074) were shown to be significantly associated with worse progression-free survival. At multivariate analysis, adenocarcinoma histotype (HR: 3.845, 95% CI: 2.980–73.592, *p* = 0.001) and incomplete cytoreduction (CC2-3) (HR: 6.747 95% CI: 1.287–35.373, *p* = 0.024) were shown to significantly influence progression-free survival (Table 5).

**Table 5:** Progression free survival analysis.

	**Univariate analysis**			**Multivariate analysis**		
**Variable**	**HR**	**95% CI**	***P***	**HR**	**95% CI**	***P***
**Gender**						
Female						
Male	1.919	0.376–9.797	0.433			
Age (years)	1.014	0.954–1.077	0.658			
BMI	1.021	0.873–1.195	0.792			
**ECOG**						
0						
≥1	0.551	0.120–2.521	0.442			
**Appendiceal histotype**						
LAMN						
Adenocarcinoma	10.986	2.555–47.238	**0.001**	3.845	2.980–73.592	**0.001**
**Prior surgical score (PSS)**						
0–2						
3	0.575	0.282–1.181	0.132			
**PSOGI classification**						
Acellular mucin						
Low-grade PMP(DPAM)						
High-grade PMP(PMCA)	9.126	2.385–34.914	**0.001**	1.529	0.147–15.857	0.722
**Preoperative chemotherapy**						
No	–	–	–			
Yes	2.741	0.552–13.603	0.217			
PCI score	1.051	0.950–1.164	0.333			
**HIPEC drugs**						
Oxaliplatin	–	–	–			
Mitomycin	1.47	0.345–6.263	0.602			
**Completeness of cytoreduction (CC)**						
0						
1						
3-Feb	4.985	0.855–29.069	**0.074**	6.747	1.287–35.373	**0.024**
**Major postoperative complications**						
*No (Clavien Dindo 0–2)*						
*Yes (Clavien Dindo 3–5)*	0.045	0.015–8.267	0.717			

*BMI, body mass index; LAMN, low-grade appendiceal mucinous neoplasm; DPAM, disseminated peritoneal adenomucinosis; PMCA, peritoneal mucinous carcinomatosis*.

*Bold value indicates significative variables and variable included in multivariable analyses*.

Overall survival was shown to be correlated to ECOG score ≥ 1 (HR: 9.621, 95% CI: 1.053–87.868, *p* = 0.045) and to CC score 2-3 (HR: 3.768, 95% CI: 1.161–12.231, *p* = 0.027) at univariate analysis. Multivariate analysis identified completeness of CC score 2-3 (HR: 3.754, 95% CI: 1.157–12.182, *p* = 0.028) as the only independent predictor of worse overall survival (Table 6).

**Table 6:** Overall survival analysis.

	**Univariate analysis**			**Multivariate analysis**		
**Variable**	***HR***	***95% CI***	***P***	***HR***	***95% CI***	***P***
**Gender**						
Female						
Male	6.083	0.311–11.049	0.234			
Age (years)	1.069	0.929–1.231	0.349			
BMI	1.706	0.892–3.263	0.107			
**ECOG**						
0						
≥1	9.621	1.053–87.868	**0.045**	3.304	0.131–83.606	0.469
**Appendiceal histotype**						
LAMN						
Adenocarcinoma	0.04	0.015–23.849	0.725			
**Prior surgical score (PSS)**						
0–2						
3	0.061	0.015–23.588	0.357			
**PSOGI classification**						
Acellular mucin						
Low–grade PMP						
High–grade PMP	1.459	0.160–13.285	0.738			
**Preoperative chemotherapy**						
No						
Yes	7.348	0.453–11.170	0.161			
PCI score	1.228	0.994–1.518	0.057			
**HIPEC drugs**						
Oxaliplatin						
Mitomycin C	3.461	0.015–37.8	0.711			
**Completeness of cytoreduction (CC)**						
0						
1						
3–Feb	3.768	1.161–12.231	**0.027**	3.754	1.157–12.182	**0.028**
**Major postoperative complications**						
No (Clavien Dindo 0–2)						
Yes (Clavien Dindo 3–5)	2.247	0.015–7.458	0.815			

*BMI, body mass index; LAMN, low-grade appendiceal mucinous neoplasm; DPAM, disseminated peritoneal adenomucinosis; PMCA, peritoneal mucinous carcinomatosis*.

*Bold value indicates significative variables and variable included in multivariable analyses*.

## Discussion

Ourstudy provides further evidence supporting the importance of complete removal of PMP peritoneal implants. In our series, incomplete cytoreduction (CC 2-3) represents the only independent prognostic factor of worse progression-free and overall survival. These results underline the role of the surgery that we were able to achieve in 47 patients, coherently with the literature data (4). A large retrospective multicenter series of 2,298 cases by Chua et al. (5) showed that patients with incomplete cytoreduction (CC2 and CC3) had a 5-year survival rate of 24%, compared to 85% in CC0 patients and 80% in CC1 patients. Indeed, complete cytoreduction is considered as one of the most important prognostic factors for overall survival and progression-free survival after CRS and HIPEC. On the other hand, several HIPEC regimens were used in this multicenter study and, despite mitomycin was largely the most common antiblastic drug administered (77% of cases), HIPEC was not shown to be a statistically significant independent factor for OS. Of note, one-third of the cohort also received early postoperative intraperitoneal chemotherapy (EPIC) that showed a correlation with higher OS only at univariate analysis.

Similarly, the prognostic role of PMP biology and its classification has been widely recognized and during the years several pathological systems have been proposed (2, 3). Recently, the PSOGI endorsed a consensus statement on PMP classification (4). According to this new classification, in our series, the great majority of patients had acellular or low-grade mucin, with 12 % displaying a high-grade disease, in line with the rates recently reported for the validation of the PSOGI pathological classification (11). In our series, considering both DFS and OS, univariate analyses revealed a trend of worse survival in patients with high-grade disease, but this finding disappeared on multivariate analysis. This result may suggest that PMP grading could be more representative of the disease behavior in terms of early recurrence rather than on prognosis, despite conclusions from a small retrospective study must be handled with care. Conversely, our multivariate analysis confirmed a statistically significant correlation between appendiceal histology and progression-free survival, with patients having adenocarcinoma histology being at higher risk of progression as compared to LAMN. Our outcomes in terms of 5-years overall and progression-free survival were, respectively, 91 and 63% (Figure 1), comparable to previously published cohorts (12–14). A drawback of these results was the extent of surgery performed. Indeed, complete cytoreduction required multiple major surgical procedures in the majority of cases, a median operating time of more than 11 h, and nearly 3 weeks of median hospital stay.

Concerning safety, the grade III-IV complication rate of the present cohort was 10%, with 2% of postoperative deaths. In a recent series by Narasimhan et al. (15) on 140 PMP patients treated with CRS and HIPEC, the grade III-IV complications incidence was 20.6%, without postoperative mortality, while the above-mentioned paper of Chua et al. (5) reported major postoperative complications in 24% of patients and 2% treatment-related mortality. Among preoperative and procedure-related variables, none was significantly associated with major postoperative complications (Table 7), so we cannot draw any conclusions on their morbidity predictive value.

**Table 7:** Postoperative complications assessed number of surgical procedures.

**Variable**	Number of surgical procedures*				***P***
	**1**	**2**	**3**	**>3**	
**Complication grade (Clavien Dindo)**					
No complications	10	7	4	4	
Grade I-II	3	3	4	4	0.52
Grade III-IV	0	1	2	1	
**Postoperative ileus**					
No	13	9	10	8	0.252
Yes	0	2	0	1	
**Pulmunary infection**					
No	12	10	9	8	0.994
Yes	1	1	1	1	
**Postoperative bleeding**					
No	13	11	9	8	0.447
Yes	0	0	1	1	
**Abdominal collection**					
No	13	11	9	8	0.447
Yes	0	0	1	1	
**Genitourinary infection**					
No	12	11	9	8	0.75
Yes	1	0	1	1	
**Wound infection**					
No	12	10	8	9	0.509
Yes	1	1	2	0	

*Surgical Procedures: gastrectomy, colectomy, rectal resection, splenectomy, hysterectomy, small bowel resection*.

In our series, all patients except three with gross residual disease received HIPEC. Despite some evidence supporting a possible benefit of HIPEC in this specific setting, a recent panel of experts was divided on administrating HIPEC after debulking for PMP (4). We do not recommend HIPEC in these cases since possible harms may outweigh the benefits. The number of patients not receiving HIPEC was also too small and affected by selection bias to provide any insight concerning the added value of HIPEC on CRS. On this issue, a recent large retrospective study of the PSOGI registry on 1,924 patients demonstrated that CRS and HIPEC are associated with better survival than CRS alone and do not worsen the postoperative outcomes (16). Noteworthy, a more favorable prognosis was observed with cisplatin plus mitomycin HIPEC and oxaliplatin plus fluorouracil-leucovorin one, while no prognostic advantage was observed in subgroups receiving mitomycin only and or other oxaliplatin-based HIPEC.

This study is affected by several limitations: (i) the retrospective nature may have lead to under-reporting of postoperative complications; (ii) the small sample size; (iii) considering the natural history of the disease which can recur even 10 years after CRS, the short follow-up we get in our series.

Due to the rarity of the disease and the ethical limit in designing randomized trials, stronger evidence will be lacking. There is a need for adequately designed large multicenter prospective studies to properly address these sensitive issues.

## Conclusions

Pseudomyxoma peritonei, if left untreated, often leads to inoperable bowel obstruction and, ultimately to death. Complete CRS associated with HIPEC has been proven to be the mainstay of treatment. Our results are in line with the available literature. Although small, this series is yet another confirmation that an aggressive surgery is justified by the outstanding survival, also considering the rate of complications and of postoperative mortality, which appears acceptable in light of the surgical effort and of the achievable results.

## Data Availability Statement

The raw data supporting the conclusions of this article will be made available by the authors, without undue reservation.

## Ethics Statement

The studies involving human participants were reviewed and approved by Fondazione Policlinico Agostino Gemelli IRCCS Ethics Committee. The patients/participants provided their written informed consent to participate in this study.

## Author Contributions

FS, SR, and AD contributed to study conception and design. CA, MA, and FG contributed to the acquisition of data. FS, MA, and SR contributed to writing the manuscript. MA, GF, and CL contributed to the drafting of the manuscript. FS, AD, and FP contributed to critical revision. All authors have read and approved the final manuscript.

## Conflict of Interest

The authors declare that the research was conducted in the absence of any commercial or financial relationships that could be construed as a potential conflict of interest.

## Publisher's Note

All claims expressed in this article are solely those of the authors and do not necessarily represent those of their affiliated organizations, or those of the publisher, the editors and the reviewers. Any product that may be evaluated in this article, or claim that may be made by its manufacturer, is not guaranteed or endorsed by the publisher.
